# Adaptive Functioning Across Contexts: A Comparison of Parent and Self-Reported Ratings in Autistic and Non-Autistic Youth

**DOI:** 10.1007/s10803-025-06756-5

**Published:** 2025-02-28

**Authors:** Jo A. Yon-Hernández, Ana-Maria Iosif, Apurv Srivastav, Marjorie Solomon

**Affiliations:** 1 Department of Psychiatry and Behavioral Sciences, University of California Davis, MIND Institute, Davis, CA USA; 2 Department of Public Health Sciences, University of California Davis, Davis, CA USA; 3 2825 50th Street, Sacramento, CA 95817 USA

**Keywords:** Adaptive functioning, Informant discrepancies, Situational specificity, Parent-youth discrepancies, CONTEXT framework

## Abstract

Multi-informants are essential for capturing the full range of adaptive functioning abilities necessary for daily living and independence. However, discrepancies within parent-child dyads, specifically comparing parent-reports to child self-reports, can cloud interpretation from assessments and hinder support planning. This study examines discrepancies in parent-youth perceptions of adaptive functioning, focusing on the social domain, and investigates associations among parents, youth, and independent ratings, considering factors like IQ, autism severity, and parental education. The study included 132 individuals (66 autistic, 66 non-autistic) aged 16–24 years. Adaptive functioning was measured using the Adaptive Behavior Assessment System-3 across the conceptual, practical, and social domains. Agreement between reporters was assessed using paired-sample *t*-tests, intraclass-correlations, and Bland-Altman plots. Spearman’s correlations examined associations between raters, while the effects of IQ, autism severity, and parental education on discrepancies were analyzed using linear regression. Autistic self- and parent-reports showed lower adaptive functioning than non-autistic dyads. Autistic youth reported higher social and less practical adaptive skills compared to parents. Autistic self-reports in the social domain correlated significantly with independent assessment. Increased autistic symptoms were associated with greater parent-child discrepancies. This study underscores the importance of multi-informant assessments to understand the full range of adaptive functioning in autistic individuals. Discrepancies in social and practical domains highlight the need for both perspectives, because parents may not observe all behaviors and may overlook autistic individuals’ perception of support needs in the practical domain. Understanding these differences is crucial for improving supports planning and enhancing quality of life for autistic individuals.

**Supplementary Information:**

The online version contains supplementary material available at 10.1007/s10803-025-06756-5.

## Introduction

The term adaptive functioning (AF) refers to the skills needed to meet daily demands and effectively adapt to various situations (Duncan & Bishop, 2015; Kanne et al., 2011) and to be self-sufficient in real-life situations (Kanne et al., 2011; Sparrow et al., 2005). Behaviors related to AF in different contexts are typically learned from an early age and are believed to mature into early adulthood (Di Rezze et al., 2019). Research has shown that autistic individuals exhibit lower AF skills than non-autistic peers at various life stages (Franchini et al., 2018).

During adolescence, autistic individuals require parental support to develop AF skills; however, the level of support decreases compared with that in earlier years (Duncan et al., 2022). Autistic adolescents without intellectual disabilities often exhibit AF abilities that are not commensurate with their cognitive abilities (Duncan & Bishop, 2015; McQuaid et al., 2021). This gap between cognitive abilities and AF skills is expected to widen through the course of development, reflecting a growing lack of adaptation to the more complex functional challenges of adulthood (Duncan & Bishop, 2015; McQuaid et al., 2021; Yon-Hernández et al., 2023).

Adaptive functioning is often evaluated using standardized assessments, which include caregiver interviews, direct observations, and other questionnaires (Golya & McIntyre, 2018; Oakland & Daley, 2013). Examples of such measures include the Vineland Adaptive Behavioral Scale—VABS (Sparrow et al., 2005) and the Adaptive Behavior Assessment System—ABAS (Harrison & Oakland, 2015), which assess functioning across conceptual (communication, academic skills, self-direction, and problem-solving), social, and practical (self-care, personal hygiene, and safety and time management) domains. Assessing AF helps identify specific areas of strengths and challenges, allowing for more precise interventions that may be required to improve daily living skills and the overall quality of life of autistic individuals. This is especially important during the transition to adulthood because AF skills are crucial for becoming more independent and navigating increasingly complex functional demands and future success in communication, socialization, and everyday tasks (Di Rezze et al., 2019; Duncan et al., 2022; Kanne et al., 2011).

Multi-informant approaches, which incorporate parent-reports (PR), self-reports (SR), and observational data, improve data accuracy and depth (Keith et al., 2019; Mazefsky et al., 2011; Sandercock et al., 2020). Parents/caregivers provide insights into behaviors at home (De Los Reyes et al., 2015; Sandercock et al., 2020), while autistic youth offer first-hand perspectives through SRs, capturing lived experiences that may not be observable (Jaswal & Akhtar, 2018; Neuhaus et al., 2024). As individuals spend more time in external settings (e.g., school, work, social activities), reliance on PRs alone becomes problematic (De Los Reyes et al., 2019). Additionally, observational data from professionals offers an objective behavioral measure. Rather than indicating inaccuracy, discrepancies between sources provide valuable context-specific insights (De Los Reyes et al., 2019; Moore et al., 2023; Sandercock et al., 2020). However, integrating different sources of information is a complex process that requires careful consideration of the reliability and context of each source (De Los Reyes et al., 2015; Sandercock et al., 2020). Understanding discrepancies and agreement among reports and factors influencing perceptions of AF—such as age, cognitive ability, and level of autistic symptom severity—is crucial (Jordan et al., 2019).

Although no single method for the integration of multiple perspectives exists, the process should involve a nuanced approach that accounts for variability, observability, and context (De Los Reyes et al., 2015, 2019, 2023; Jordan et al., 2019). Informant discrepancies are common among youth, as different informants (e.g., parents, teachers, youth themselves) assess behavior in distinct social contexts. Traditionally, the Converging Operations and Multi-Trait Multi-Method Matrix (MTMM) paradigms have framed discrepancies as measurement confounds, such as random error or rater biases (De Los Reyes et al., 2023). However, the Operations Triad Model, introduced within the past decade, recognizes that while some informant discrepancies reflect measurement confounds, others provide crucial domain-relevant information (De Los Reyes et al., 2019, 2023). To better understand these discrepancies, De Los Reyes et al. (2023) proposed a new framework, Classifying Observations Necessitates Theory, Epistemology, and Testing (CONTEXT), which advances measurement validation by guiding researchers to (a) leverage informants that produce domain-relevant discrepancies, (b) use analytic procedures that retain these discrepancies, and (c) design studies that contain domain-relevant information.

### Understanding Discrepancies and Agreement in Rating Adaptive Behavior in Autism

Parent-teacher discrepancies in AF have been extensively studied in autistic children and youth, offering insights into inter-rater agreement and discrepancies between parents and children. Similar to the challenges De Los Reyes et al. (2023) identified in their CONTEXT approach, current studies examining parent-teacher discrepancies in autism have highlighted two primary issues. First, studies traditionally rely on single metrics (e.g., mean comparisons or correlational measures) (Jordan et al., 2019) to analyze inter-rater agreement. Second, integrating multi-informant reports, requires a nuanced approach to accurately capture the complexities of AF across different contexts, perspectives, and stages of development. Variability in AF abilities across settings contributes to discrepancies, as parents may not observe the full spectrum of their children’s behaviors in school settings, and teachers may possess different internal standards for assessing child behavior based on their more extensive training and experience working with many children (De Los Reyes et al., 2015, 2019).

Based on these challenges, studies have used various statistical approaches aligned with the CONTEXT approach to capture AF strengths and challenges in autism. Jordan et al. (2019) used multiple metrics, including paired-sample *t-*tests to identify significant differences between raters in AF domains, intraclass correlation coefficients (ICCs) to measure agreement levels between raters, and Bland-Altman plots to visually represent the agreement or disagreement patterns between the raters across AF scores (Bland & Altman, 1986; Mansournia et al., 2021). Linear regression models were used to predict how factors such as child age, intelligence quotient (IQ), language level, autism severity level, and parent education level could influence levels of agreement as well as discrepancies between them (Jordan et al., 2019; Moore et al., 2023). Reports from independent raters can also be used to validate the relative ratings of parents and children (De Los Reyes & Kazdin, 2005).

Jordan et al. (2019) examined parent-teacher discrepancies in assessing AF among autistic children (ages 6–12), finding that teachers rated practical skills higher than parents. However, child age, intellectual ability, language level, autism severity, and parental education level did not explain these discrepancies, suggesting other contributing factors. Similarly, Moore et al. (2023) found that teachers and parents provided similar AF ratings for younger children (2–11 years), except in the social domain, where parents rated their children higher. This difference may reflect teachers’ broader perspectives on AF skills in peer interactions. Parent-teacher agreement was poor (ICCs < 0.5) across social, conceptual, and practical domains, and Bland-Altman plots revealed systematic differences, particularly in social AF. Nonverbal ability, age, and autism severity contributed to these discrepancies, likely reflecting differences in observational contexts or parental rating thresholds. Using the same analytical approach, Stevens et al. (2023) found that teachers rated AF higher than parents across all domains in autistic individuals aged 2–19 years, identifying autism severity as the sole predictor of discrepancies. These studies highlight how rating differences provide domain-relevant insights across settings, aligning with the CONTEXT paradigm (De Los Reyes et al., 2019; Los Reyes et al., 2023).

Across all reviewed studies on the parent-child agreement and discrepancies, larger differences and mixed results were observed when assessing the social AF domain, aligning with literature on other social constructs in autism (Bakhtiari et al., 2021). Social functioning discrepancies are common in both clinical and non-clinical populations (De Los Reyes et al., 2023). This is particularly relevant for autistic individuals because social functioning challenges are a core characteristic of autism. Parents and youth differ in their beliefs and expectations regarding the importance of social AF skills in daily life. These beliefs are influenced by situational specificity and varying contexts in the youth’s life (De Los Reyes et al., 2019; Rankin et al., 2016). Autistic adolescents often rate their social skills higher than their parents, highlighting a pronounced discrepancy (Bakhtiari et al., 2021; Lerner et al., 2017).

The current manuscript is the first to examine the associations between PRs and SRs in ratings of AF among highly able autistic adolescents and young adults. We used multiple assessment forms and drew heavily on the literature on parent and teacher ratings of AF (Jordan et al., 2019; Moore et al., 2023; Stevens et al., 2023). We also incorporated the CONTEXT model (De Los Reyes et al., 2023), which posits that discrepancies in informant ratings reflect meaningful variations across developmental and situational contexts (De Los Reyes et al., 2019; Jepsen et al., 2012), to guide hypothesis generation. We first hypothesized that PR-SRs discrepancies, across most methods, would be most pronounced in the social domain, as social AF skills occur outside parental observation in individuals aged 16 to 24. We also expected PRs to correlate weakly with independent ratings than SRs, which are likely to improve with age, self-awareness, and communication skills (Uddin et al., 2013; Vijayakumar et al., 2018). Second, we hypothesized that discrepancies between reporters are associated with factors such as IQ, autism severity, and mother education level, consistent with findings on parent-teacher agreement (Stevens et al., 2023).

## Methods

The present research was conducted at the University of California, Davis MIND Institute, as part of a 5-year cohort-sequential study (Prinzie & Onghena, 2005) on cognitive control in the greater Sacramento area. This longitudinal research project, Cognitive Control in Autism (CoCoA), investigated cognitive development and outcomes during the pivotal transition to young adulthood, with participant assessments at 3 time points. The complete cohort included 176 participants (88 non-autistic and 88 autistic). The inclusion criteria for the present study required the availability of both PR and SR at the same time point. If data from multiple time points were available, the earliest was selected. Thus, the analyzed sample comprised 132 participants: 66 in the non-autistic group and 66 in the autistic group.

### Participants

All participants were ages 16–24 years and had a Full-Scale Intelligence Quotient (FSIQ) of ≥ 70 as evaluated using the Wechsler Abbreviated Scale of Intelligence-II (Wechsler, 2011). The IQ criterion was set at ≥ 70 based on the inclusion criteria of a prior study to ensure that participants had the cognitive ability required for completing assessments. Autistic participants were evaluated by licensed clinicians and met the autism criteria using both the Diagnostic and Statistical Manual of Mental Disorders-5 (DSM-5) (American Psychiatric Association, 2013) and the Autism Diagnostic Observation Schedule-2 (ADOS-2) (Lord et al., 2012). Non-autistic participants had no history of neurodevelopmental disorders and no first-degree relatives with a diagnosis of autism. Furthermore, they did not meet the autism spectrum criteria based on the Social Communication Questionnaire (SCQ) (Rutter et al., 2003). Participants were matched according to age to ensure comparability across groups. However, because of parent and child data availability, only a subset of participants could be matched for IQ. This indicates that although age matching was consistent across all participants, IQ matching was limited and was not achieved for the entire sample. We acknowledge that this is a limitation of our study. The study received approval by the University of California, Davis Institutional Review Board (#254439-48).

### Measures

#### Autism Diagnostic Observation Schedule 2 (ADOS-2) (Lord et al., 2012)

The ADOS-2 is a semi-structured interview conducted by a licensed clinical psychologist trained in reliability in administration and scoring. The tool assesses autism spectrum disorder symptoms in 2 domains: social communication and interaction and restricted/repetitive behaviors. To participate in this study, autistic participants were required to meet the ADOS-2 threshold score corresponding to their chronological age and language development, thereby confirming the diagnostic criteria. ADOS-2 provides a calibrated severity score (CSS) to compare symptom severity across assessment modules.

#### Wechsler Abbreviated Scale of Intelligence, 2nd Edition (WASI-II) (Wechsler, 2011)

The WASI-II is a brief assessment of cognitive abilities. It measures verbal comprehension using vocabulary and similarities subtests, thus producing the Verbal Comprehension Index (VCI). Perceptual Reasoning Index (PRI) was measured using block design and matrix reasoning subtests. These scales produce the Full-Scale IQ (FSIQ) score, an overall estimate of general intellectual functioning.

#### Adaptive Behavior Assessment System- Third Edition (ABAS-3) (Harrison & Oakland, 2015)

The ABAS-3 is a comprehensive questionnaire designed to assess AF throughout life. The ABAS-3 has different forms; we used the Adult Form to gather assessments from the non-autistic and autistic participants and the Parent Form for the caregivers. On the ABAS-3, raters indicate the frequency of an individual’s performance of each activity on a 4-point response scale. The ABAS-3 yields a General Adaptive Composite (GAC) score encompassing 3 broad adaptive domains: conceptual, social, and practical. The conceptual domain includes the skill areas of communication, functional academics, and self-direction. The social domain includes Social and Leisure skill areas. Finally, the practical domain includes self-care, home living, community use, health and safety, and work. These skills encompass daily living and self-care in various settings, such as home, workplace, community, and classroom, and address personal and health needs. Higher ABAS-3 scores indicate stronger AF skills. Scores above 120 are considered high; scores between 120 and 111 are considered above average; scores ranging from 110 to 91 are considered average; scores between 90 and 81 are considered below average; scores between 80 and 71 are considered low; and scores below 70 are considered extremely low. It is essential to note that the ABAS-3 is better suited to individuals with higher intellectual abilities because it covers more advanced adaptive skills. This study used standard scores for the GAC, conceptual, social, and practical composites as dependent variables.

#### Global Functioning: Social Scale (GF: Social) (Carrión et al., 2019; Cornblatt et al., 2015)

We used the GF: Social Scale to provide an independent measurement of youth social functioning. The GF: Social scale evaluates the quantity and quality of peer relationships, levels of peer conflict resolution, age-appropriate intimate relationships, and involvement with family members. Special emphasis is placed on age-appropriate social contacts and interactions beyond the family circle, particularly social withdrawal and isolation. Scores range from 1 to 10, with 10 indicating superior functioning and 1 indicating significant difficulties. The GF: Social was designed to be used by experienced clinicians to summarize previously collected data or as a direct interview guided by the accompanying probes. The GF: Social scale has been validated in youth at high risk for schizophrenia (Cornblatt et al., 2015). In this study, we adapted the scale to better capture autism-specific social challenges and ensure age-appropriateness. The revisions included refining prompts and anchors to emphasize social initiation, reciprocal social communication, and social responses. Furthermore, modifications include integrating technology-based interactions such as social media and online communication and shifting the focus from generalized clinical symptoms (e.g., paranoia) to autism-specific social difficulties. Expert consensus informed these adaptations to enhance their relevance and usefulness for the study population. A detailed description of the adaptation process is included in the Supplemental Information. We used the GF: Social scale as the independent variable.

### Analysis

Paired data for PR and SR on the ABAS-3 were used in the analysis. Descriptive statistics were calculated to summarize participant characteristics and clinical features. We used independent two-sample *t*-tests to assess group differences in participant characteristics (age, IQ, SCQ) and across all AF domains.

To assess cross-informant differences according to the type of report (PR vs. SR) on the AF domains measured by the ABAS-3, paired-sample *t*-tests and effect size estimates (Cohen’s *d*) were calculated. The agreement and discrepancies between ratings were analyzed using both ICC and the Bland-Altman plots. We chose ICC over simple correlations because ICC measures the degree of correlation and provides a more comprehensive assessment of agreement between ratings (Koo & Li, 2016). ICC and Bland-Altman together offer a robust approach for evaluating agreement between raters in research involving subjective assessments. While ICC quantifies overall agreement and consistency among raters, it does not provide information about the direction and magnitude. Bland-Altman plots complement ICC by visually representing disagreement patterns and extent, facilitating the identification of systematic biases and outliers (Bland & Altman, 1986; Mansournia et al., 2021). To indicate the level of agreement, we used Cicchetti (1994): an ICC of > 0.9 is classified as excellent agreement; an ICC between 0.75 and 0.9 as good agreement; 0.5 and 0.75 as moderate agreement; and < 0.5 as poor agreement. Perfect agreement between parent and child ratings would mean that the mean difference between them is zero, as illustrated by the Bland-Altman plots (Bland & Altman, 1986; Mansournia et al., 2021).

Next, we evaluated the associations between independent reports (GF: Social scale) and the ABAS-3 scores of parents and children using correlations. We calculated Spearman’s rank order correlation coefficients because of the non-normal distribution of the data. The analysis was conducted for both groups. The interpretation of Spearman’s correlation coefficients was as follows: 0 to 0.39 – weak; 0.40 to 0.69 – moderate; > 0.70 – strong (Schober et al., 2018). Statistical significance was set at *p* <.05, and all *p*-values were two-tailed. Lastly, we conducted linear regression models to explore the role of the child’s age, IQ (VCI and PRI), ADOS-2 CSS (autism severity symptoms for the autistic individuals), and mother’s education level are associated with the discrepancies between parent-child reports on the ABAS domains (GAC, Social, Conceptual, and Practical) in both autistic and non-autistic groups. Analyses were implemented in SAS Version 9.4 (2021) and R Core Team (2021).

## Results

The Supplemental Materials provide a detailed description of the sample demographics and other clinical features, as well as the between-group comparisons (autistic vs. non-autistic participants) of AF skills. These comparisons revealed significant group differences, with autistic participants demonstrating lower adaptive functioning skills, with the average score being one standard deviation below that of non-autistic participants.

### Cross-Informant Group Comparisons

Results of paired-sample *t*-tests showed that in the autistic group, a significant difference was observed between PR and SR for the social composite (PR > SR), *t*(65) = -4.51, *p* =.001. The effect size was medium (*d* = − 0.60). No other statistically significant differences in PR vs. SR were observed in either group in any AF domain (see Table 1).

**Table 1:** Paired samples statistical analysis of parent and self-reported ABAS-3 composites

		Mean	SD	Mean difference	Paired difference (PR vs. SR)		
					*t*	*p*-value	Cohen’s *d*
**Non-Autistic Group**							
Pair 1	GAC PR	105.2	10.9	− 1.47	− 0.75	0.46	− 0.09
	GAC SR	106.9	13.9				
Pair 2	Conceptual PR	105.8	10.9	− 0.58	− 0.27	0.79	− 0.03
	Conceptual SR	106.5	14.1				
Pair 3	Social PR	103.2	10.2	− 2.39	− 1.51	0.14	− 0.19
	Social SR	105.6	10.4				
Pair 4	Practical PR	105.8	11.3	− 0.12	− 0.06	0.95	− 0.01
	Practical SR	106.2	14.4				
**Autistic Group**							
Pair 1	GAC PR	80.8	11.4	− 2.11	− 1.17	0.25	− 0.14
	GAC SR	82.9	15.3				
Pair 2	Conceptual PR	84.0	10.7	− 0.47	− 0.24	0.81	− 0.03
	Conceptual SR	84.5	16.4				
Pair 3	Social PR	78.4	11.6	− 7.65	− 4.51	0.01^*^	− 0.60
	Social SR	86.0	13.3				
Pair 4	Practical PR	83.1	12.8	0.79	4.61	0.68	0.05
	Practical SR	82.4	15.4				

*Note*** SD**: Standard Deviation; **GAC**: Global Adaptive Composite; **PR**: Parent-Report; **SR**: Self-Report

^*^Significant at *p* <.01 level

### Parent-Youth Reliability Estimates

To assess the agreement between the PR and SR, an ICC analysis was performed. According to the norms established by Cicchetti (1994), both groups showed poor agreement between the raters on all AF domains, given that all were below 0.5 (see Table 2). However, ICC values for the autism group were closer to reaching the threshold for moderate agreement compared with the non-autistic group. The highest agreement in the autism group was observed for the social composite.

**Table 2:** Intraclass correlation analysis of the agreement between AF domains between parent and youth

	ICC (95% CI)	Value	df1	df2	*p*-value
***Non-Autistic Group***					
GAC	0.19 (-0.06, 0.41)	1.46	65	65	0.065
Conceptual	0.08 (-0.17, 0.31)	1.17	65	65	0.266
Social	0.23 (-0.01, 0.45)	1.61	65	65	0.029
Practical	0.27 (0.03, 0.48)	1.72	65	65	0.015
***Autistic Group***					
GAC	0.41 (0.19, 0.59)	2.40	65	65	< 0.001
Conceptual	0.36 (0.13, 0.55)	2.10	65	65	0.002
Social	0.47 (0.26, 0.64)	2.80	65	65	< 0.001
Practical	0.40 (0.17, 0.58)	2.32	65	65	< 0.001

*Note*** ICC**: Intra-Class Correlation Coefficient; **df**: degrees of freedom; **CI**: Confidence of Interval; **GAC**: Global Adaptive Composite

We used Bland-Altman plots to examine parent-youth agreement. These plots display the differences between parent and youth ratings (PR - SR) against their average score ([PR + SR]/2). On the graph, the vertical (*y*) axis shows the score differences, and the horizontal (*x*) axis shows the average of the ratings. Each plot includes a solid line representing the average difference between parent and youth ratings (mean difference) and dashed lines indicating the 95% limits of agreement, which define the range of agreement above and below the mean. These limits are graphically shown as the upper limit (UL) and lower limit (LL). Good agreement is shown when the mean difference is close to zero and the limits of agreement are narrow, typically within ± 5 points.

**Fig. 1:**
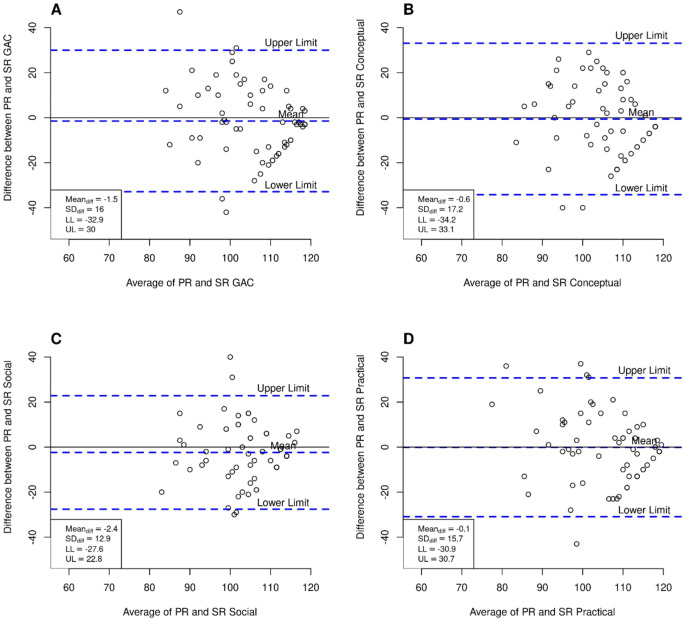
Bland-Altman Plots showing the agreement between parent and youth in the non-autistic group. In each panel, the solid line represents the mean difference (bias), and the dashed lines indicate the 95% limits of agreement (LoA) between parent and self-report ratings. Individual points represent paired differences, and the vertical spread reflects variability. Panel **A** (GAC: global adaptive composite), Panel **B** (conceptual domain), Panel **C** (social domain), and Panel **D** (practical domain)

Figure 1 shows the Bland-Altman analysis for the non-autistic group, with each panel representing a specific domain of the ABAS-3. The analysis reveals limited agreement between parent and child ratings, with parents consistently rating their children lower than the children rated themselves. The wide limits of agreement reflect considerable variability in the differences between parent and child assessments, indicating inconsistency and reduced reliability in the paired scores.

**Fig. 2:**
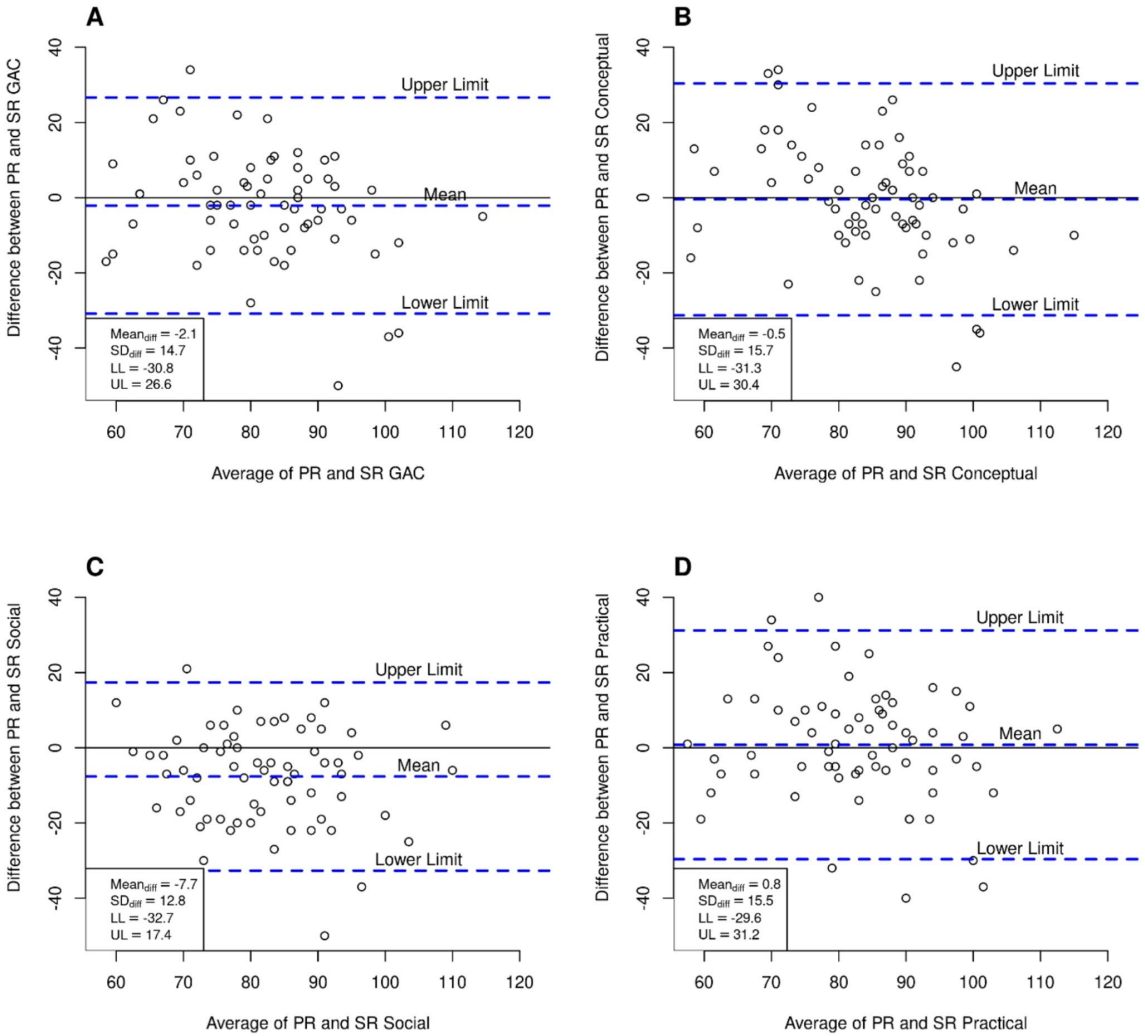
Bland-Altman Plots showing the agreement between parent and youth in the autistic group.. In each panel, the solid line represents the mean difference (bias), and the dashed lines indicate the 95% limits of agreement (LoA) between parent and self-report ratings. Individual points represent paired differences, and the vertical spread reflects variability. Panel **A** (GAC), Panel **B** (conceptual domain), Panel **C** (social domain), and Panel **D** (practical domain)

Figure 2 presents the analysis conducted for the autism group. Similarly to the non-autistic group, Panel A (GAC), B (conceptual domain), and C (social domain) indicate limited agreement between parent and youth ratings, with parents rating lower these AF skills than their children. The wide limits of agreement reflect considerable variability in the differences between parent and child assessments, indicating inconsistency and reduced reliability in the paired scores. The largest discrepancies between PR and SR were observed in Panel C for the social AF domain, with a mean difference of -7.65 (LL -32.69, UL 17.38). This discrepancy between ratings is consistent with the results of the *t*-tests.

Lastly, Panel D (practical domain) shows a different trend, in which parents rated higher their child’s practical AF skills. Nonetheless, the analysis indicated limited agreement between the 2 assessment modalities, as indicated by a mean difference of 0.79 (LL -29.65, UL 31.23).

### Associations Between Parent and Self-Reports, and an Independent Rater

A Spearman's rankorder correlation analysis was conducted to examine the relationship between PR and SR on the ABAS-3 and an independent rater assessment using the GSGR scale. The initial examination revealed a monotonic relationship observed through scatterplot visualization in both groups. For the non-autistic group, no significant correlations were found between the PRs and independent rater assessments, nor between the SRs and independent rater assessments (see Table 3). In contrast, for the autistic group, significant correlations were observed between parent and self-ratings on all ABAS-3 composites with the GS scale. Autistic PR correlations ranged from small (0.25) to medium-sized correlations (0.48), with the highest being Social PR with GS (Table 3). Social PR was also significantly correlated with GR (0.25). For autistic self-ratings and the independent assessment of GS correlations ranged from medium correlations (0.40) to large (0.50), with Social SR (0.50) having the largest correlation with GS (see Table 3).

**Table 3:** Spearman’s rankorder coefficients for the correlations between the ABAS-3 composites from parent and self-assessment and the GF: Social Scale

Variable	Non-autistic group		Autistic group	
	*n*	GF: Social	*n*	GF: Social
1. GAC PR	*66*	0.06	*66*	0.38^**^
2. Conceptual PR	*66*	0.05	*66*	0.25^*^
3. Social PR	*66*	0.13	*66*	0.48^**^
4. Practical PR	*66*	− 0.01	*66*	0.27^*^
5. GAC SR	*66*	0.18	*66*	0.46^**^
6. Conceptual SR	*66*	0.12	*66*	0.42^**^
7. Social SR	*66*	0.15	*66*	0.50^**^
8. Practical SR	*66*	0.16	*66*	0.40^**^

**GAC**: Global Adaptive Composite; **PR**: Parent-Report; **SR**: Self-Report; **GF**: Global Functioning

^**^The correlation was significant at the 0.01 level (2-tailed)

^*^The correlation was significant at the 0.05 level (2-tailed)

### Correlates of Parent-Youth Discrepancies

We investigated several variables to determine their association with parent-youth rating differences in the GAC and 3 adaptive domains in each group. The variables included in the linear regression models were the child’s age, IQ (VCI and PRI), autism severity level (ADOS-2 CSS, only for the autistic group), and mother education level (measured in years).

The model for GAC in the autistic group showed statistical significance, *F*(5, 59) = 3.79, *p* =.005, explaining 24.3% of the variance (R^2^ = 0.243, adjusted R^2^ = 0.179). ADOS-2 CSS emerged as the only significant association (β = -4.65, *p* =.001), with higher autism symptom severity associated with lower differences between informants (PRs - SRs).

The model for Social scores in the autistic group reached significance, *F*(5, 59) = 3.65, *p* =.006, with an R^2^ of 0.236 (adjusted R^2^ = 0.172). We found that both ADOS-2 CSS (β = -3.75, *p* =.001) and VCI (β = − 0.33, *p* =.011) significantly associated with lower discrepancies between the reporters (PRs - SRs).

The Practical scores model was also significant, *F*(5, 59) = 2.77, *p* =.026, accounting for 19.0% of the variance (R^2^ = 0.190, adjusted R^2^ = 0.121). ADOS-2 CSS (β = -3.88, *p* =.002) emerged as the only significant association (PRs - SRs).

The Conceptual scores model achieved significance, *F*(5, 59) = 4.08, *p* =.003, explaining 25.7% of the variance (R^2^ = 0.257, adjusted R^2^ = 0.194). ADOS-2 CSS (β = -5.25, *p* <.001) and VCI (β = − 0.29, *p* =.046) both contributed significantly to the model, with higher scores associated with lower discrepancies (PRs - SRs).

The models for the non-autistic group did not reach significance for any composite (all *p* >.05). The low R^2^ values suggest that the associations had minimal explanatory power in this group.

## Discussion

The current study sought to address gaps in the understanding of AF by examining discrepancies between PRs and SRs during the transition to adulthood in both autistic and non-autistic youths without intellectual disability. In line with the CONTEXT framework, which highlights that discrepancies in informant reports often reflect situational specificity, our findings revealed that autistic and non-autistic parents and youth consistently disagreed across all AF domains. These discrepancies were particularly pronounced in the social domain, where autistic, but not non-autistic, youth rated social AF abilities significantly higher than their parents. ICCs and Bland-Altman plots revealed that parent-child agreement was generally higher in dyads with autistic youth compared to those with non-autistic youth. Additionally, Bland–Altman plots from the practical domain showed that autistic youth rated their AF abilities lower than their parents did, in contrast to the social domain, where they rated their abilities higher. Notably, unlike the non-autistic group, reports from autistic youth and their parents showed significant correlations with an independent rating of social functioning, GF: Social, with the autistic youth showing the highest correlation between social AF on this independent measurement. Finally, in the autistic group, greater discrepancies between PRs and SRs were associated with higher levels of autistic symptoms and lower VCI scores, particularly in the Social and Conceptual domains.

A growing body of research has demonstrated that SRs from autistic youth provide insights more closely aligned with objective measures than PRs (Keith et al., 2019; Santore et al., 2020). This was also observed in the current study for autistic individuals but not for non-autistic individuals. While SRs and PRs reflect different perspectives and contexts, the alignment of SRs with independent assessments in this study highlights the unique and complementary value of SRs, particularly in capturing social and practical AF skills. This supports the idea that discrepancies are not merely measurement confounds but reflect domain-relevant information shaped by each informant’s specific observational context (De Los Reyes et al., 2023; Lerner et al., 2017). The nature of our sample, which consisted of cognitively able individuals with a relatively high degree of independence given their advanced age, may help explain parent-youth discrepancies. This reinforces the potential value of SRs relative to capturing relevant and accurate information. Incorporating SRs into assessments can lead to better-informed decisions and ultimately improve support strategies implemented for autistic individuals. Our results suggest that situational specificity is key to interpreting discrepancies because it reflects the differing contexts in which adaptive behaviors are observed and can provide nuanced insights into the lived experiences of autistic individuals.

The relationship between autism severity and parent-youth rating discrepancies was another crucial finding of this study. Higher levels of autism severity corresponded to the differences in ratings, indicating that challenges associated with more severe symptoms may intensify differences in perceptions of AF in parents and youth. This finding is consistent with that of previous studies on parent-teacher ratings among younger individuals (Moore et al., 2023; Stevens et al., 2023). The severity of autistic symptoms is often correlated with greater difficulties in AF (Jordan et al., 2019; Stevens et al., 2023). Severe symptoms, such as communication challenges, repetitive behaviors, and social deficits, can significantly affect daily functioning, leading parents to rate AF lower (Jordan et al., 2019; Stevens et al., 2023). Effective communication involves not only verbal comprehension but also other language abilities, such as expressive and receptive skills, pragmatic language, and the ability to adapt communication to various social and contextual demands. Our findings suggest that higher VCI may help foster better understanding between parents and youth. However, while VCI may serve as a proxy for certain cognitive-linguistic skills, it does not necessarily translate to effective communication in all contexts. Although previous studies have not found a correlation between VCI and parent-child discrepancies (e.g., Jordan et al., 2019; Stevens et al., 2023), our findings show relevance to reducing discrepancies in AF ratings. The CONTEXT model suggests that these discrepancies may provide domain-relevant information about how the severity of autistic symptoms influences the perception of functioning among parents and youth in different settings. The CONTEXT model further suggests that the variability in symptoms across different settings and situations can cause discrepancies because parents may not observe the full scope of their children’s behaviors (De Los Reyes et al., 2023), and these differences reflect the diverse contextual factors that shape each informant’s perspective (De Los Reyes et al., 2015, 2019, 2023; Lerner et al., 2017; Rankin et al., 2016). Interestingly, the child’s age and the mother’s education were not correlated with the discrepancies found in the AF domains assessed in this study. Moreover, the findings suggest that parents may underestimate the AF skills of youths with more pronounced symptoms, which could lead to their inhibition of child independence and self-determination (Jordan et al., 2019; Moore et al., 2023). This highlights the importance of involving youth directly in the assessment process to ensure that their perspectives, reflective of their unique situational contexts, are adequately considered.

In general, the results indicated that levels of agreement between parents and youth, as indexed by different measurements, were relatively low. Analysis using ICCs showed poor agreement across all AF domains in both autistic and non-autistic groups. Bland-Altman plots further corroborated these findings, although they did illustrate domain-specific discrepancies in the practical domain. Here, while the parents of autistic youth consistently rated their children’s AF skills lower than their children’s self-ratings, parents rated their children’s skills higher in the practical domain. These findings suggest that discrepancies arise from differences in the observational context and perspective of each rater, translating into domain-specific information. Parents may rate their children’s AF skills differently because they focus on distinct contexts—such as structured environments at home versus less structured social settings—where their opportunities to observe certain behaviors are limited. This contextual understanding helps explain why discrepancies are most pronounced in domains where parents have less direct observation, as noted by Jepsen et al. (2012); De Los Reyes et al. (2019); De Los Reyes et al. (2023). Nonetheless, it is important to note that although we observed considerable variability between parent and child ratings, there were individual dyads in the autistic group that showed higher agreement compared to the non-autistic dyads. The reason for this is unclear but may be related to specific parent-child dynamics, contextual factors, or even the nature of the biases themselves, which were not fully captured in our study. Further research is required to explore these aspects in greater detail. Moreover, these findings support the CONTEXT framework’s suggestion that such discrepancies reflect the differing contexts in which behaviors are observed, with autistic youth possibly rating higher their social skills based on limited or distinct social interactions compared to their parents’ broader observational context. Lastly, it is important to understand why different statistical tests may yield seemingly conflicting results and their implications when interpreting discrepancies in AF. Paired *t*-tests detect specific mean differences between reporters (parents and youth) within a domain, such as significant discrepancies observed in the social AF domain. These tests highlight systematic differences in ratings. In contrast, ICCs and Bland-Altman plots measure overall agreement across all data points, providing a broader view of consistency between reporters. The larger discrepancies observed in the social domain revealed by paired *t*-tests may reflect the subjective and context-dependent nature of social interactions. In contrast, the relatively higher agreement observed in autistic dyads through ICCs and Bland-Altman plots suggests a pattern of agreement that, while still poor, was better than that observed in non-autistic dyads. These complementary methods provide nuanced insights into parent-youth agreement patterns, emphasizing the need for interventions that address both domain-specific and overall discrepancies to support the unique needs of autistic youth and their families.

Although this study provides valuable insights, several limitations should be considered when interpreting the results. We included only able and unmedicated participants in the study due to the original CoCoA study aims, which may limit the generalizability of the findings to a broader population. It is important to acknowledge that while we used gold-standard research-based measures to enroll autistic participants, we did not collect the more extensive developmental history, teacher assessment, and parent interviews that one would want to obtain to make a comprehensive diagnosis in clinical practice. This may have caused us to be overly inclusive or to miss individuals in our participant group. Additionally, the ADOS-2 CSS used in this study was designed to establish thresholds for autism risk and track individual trajectories within a range over time; however, it has limitations as a measure of overall autism symptom severity, particularly in the case of between-group comparisons. However, CSS was not used for between-group comparisons in this study. The inability to match groups on IQ is another limitation that may have influenced between-group comparisons. A group of autism specialists has adapted the GF: Social scale, but it has not yet been fully validated in an autistic sample. Future research should replicate the findings with larger samples and alternative assessment tools to further validate the results. Longitudinal designs should also be considered to better examine how parent-youth discrepancies in AF evolve over time and impact long-term outcomes and potentially help identify critical periods when interventions might be most effective. Incorporating additional independent measures of AF, such as direct observations and teacher reports, could provide a more comprehensive assessment. Additionally, future studies should investigate the potential impact of cultural factors and parental expectations on parent-youth discrepancies in AF assessments.

In conclusion, this study underscores the need for comprehensive assessments incorporating multiple informants to fully understand discrepancies and agreements in AF reports. By considering multiple perspectives, we can achieve a more accurate and holistic understanding of AF that ensures that the diverse experiences and viewpoints of the reporters are acknowledged with the ultimate goal of more precisely targeting more effective and personalized support strategies during the pivotal transition to adulthood. In particular, the CONTEXT paradigm allows us to comprehensively interpret informant discrepancies not merely as errors but as a meaningful reflection of the diverse perspectives and contexts each informant brings in in terms of strengths and areas where autistic youth need assistance using AF skills. More importantly, we can conclude that incorporating SRs into assessments of AF allows for a better understanding of the youth’s own perspectives and abilities, leading to treatment strategies that are better aligned with their actual needs and experiences, thereby improving outcomes during the critical transition to adulthood.

## Electronic supplementary material

Below is the link to the electronic supplementary material.

Supplementary Material 1
