# Supplementary material for: Adaptive Functioning Across Contexts: A Comparison of Parent and Self-Reported Ratings in Autistic and Non-Autistic Youth
Source: J Autism Dev Disord. 2025 Feb 28;56(7):2839–50. doi: 10.1007/s10803-025-06756-5 (PMC12702463; doi:10.1007/s10803-025-06756-5)
Supplement: Supplementary file 1 — Supplementary Material 1 [file 10803_2025_6756_MOESM1_ESM.docx]

**Supplementary Information**

**Title:** Adaptive functioning across contexts: A comparison of parent and self-reported ratings in autistic and non-autistic youth

**Journal:** Journal of Autism and Developmental Disorders

**Measures**

*Global Functioning: Social Scale (GF:Social) (Carrión et al., 2019; Cornblatt et al., 2015)*

As part of the adaptation of the GF:Social scale for this study, we refined prompts and anchors to better capture autism-specific social challenges and ensure age-appropriateness. The revisions emphasized key aspects of social functioning, including social initiation, reciprocal social communication, and social responses. Additional modifications to integrate technology-based interactions, such as social media and online communication. Furthermore, the focus was shifted from generalized clinical symptoms (e.g., paranoia) to autism-specific social difficulties.

Expert consensus informed these adaptations to enhance the scale’s relevance and utility for the study population. Specific questions to guide the rating of the GF:Social scale are provided below. Ratings should consider changes in social functioning over the previous year (capturing both highest and lowest levels of functioning) as well as current functioning within the past month. Below, additional prompts included in this adaptation are highlighted in **bold**.

1. Tell me about your social life. Do you have friends?

2. Are they casual or close friends?

- If casual—Are they school or work friends only? **How long have you known them? What kind of things do you like doing together?**
- If close—**How did you meet? How long have you known them? What kind of things to you like doing together?**
- **What does being a close friend mean to you?**

3. How often do you see friends? Do you see them outside of work/school? When was the ‘‘last time’’

you saw one of your friends outside of work/school? (Attempt to determine ‘‘actual’’ amount of social contact vs perceived amount of social contact.)

4. Do you usually initiate contact or activities with friends or do they typically call or invite you? Do you ever avoid contact with friends? **If so, why?**

5. Do you ever have problems/falling outs/**disagreements with friends? Did you make up and how did that go?**

6. Are you dating or interested in dating? (Alter as needed to assess age-appropriate intimate relationships)? **Have you ever had a serious relationship? How long did it last? Do you think you want to have a long-term partner one day?**

7. Do you spend time with family members (at home)? How often do you communicate with them? Do you ever avoid contact with family members?

**8. Please help me to understand your use of the internet. Do you text or e-mail with your friends and family? If so, who do you use these with and how often? Do you use live stream chatting?**

**9. Please help me to understand your use of social media. Do you use social media? Which outlets do you use? How often do you use them? What do you like to do on social media?**

Additionally to the prompt additions, we also proposed the following adaptations to the original GF:Social rating scale (Cornblatt et al., 2007).

| GF: Social Rating Scale | |
| --- | --- |
| **Score** | **Superior social/interpersonal functioning** |
| **10** | **Original:** Superior functioning in a wide range of social and interpersonal activities. Frequently seeks out others and has multiple satisfying interpersonal relationships, including multiple close and casual friends. Is sought out by others because of his or her many positive qualities. Age-appropriate involvement in intimate relationships.  **Proposed:** Interested and involved in a wide range of interpersonal activities in the community including both close reciprocal friendships and acquaintances. The individual is sought out by others because of his or her many positive social qualities. The individual does not appear to be socially awkward. The individual shows age-appropriate involvement in intimate relationships and has strong social problem-solving skills. Individual may have internet communication including gaming, text-based and verbal contact, face-to-face communication, and social media. |
|  | **Above average social/interpersonal functioning** |
| **9** | **Original:** Good functioning in all social areas, and interpersonally effective. Interested and involved in a wide range of social and interpersonal activities, including both close and casual friends. Age-appropriate involvement in intimate relationships. No more than everyday interpersonal problems or concerns (e.g., an occasional argument with spouse, girlfriend/boyfriend, friends, coworkers, or classmates). Able to resolve such conflicts appropriately.  **Proposed:** Interested and involved in a wide range of interpersonal activities including both close reciprocal friendships and acquaintances. Regularly leaves home without prompting. Has no difficulty initiating interactions with new people. If, very infrequently, social interactions with community members, friends, and intimate partners are awkward, the individual has sufficient insight to learn and to make things right if necessary and it does not interfere with relationships. The individual shows age-appropriate engagement in intimate partnerships. Individual may have internet communication including gaming, text-based and verbal contact, face-to-face communication, and social media. |
|  | **Good social/interpersonal functioning** |
| **8** | **Original:** Some transient mild impairment in social functioning. Mild social impairment is present, but transient and expectable reactions to psychosocial stressors (e.g., after minor arguments with spouse, girlfriend/boyfriend, friends, coworkers, or classmates). Has some meaningful interpersonal relationships with peers (casual and close friends), and/or age-appropriate intimate relationships. Infrequent interpersonal conflict with peers.  **Proposed:** Mild and transient social awkwardness and anxiety is present only when there are intermittent stressors (e.g. losing a relative, friend moving, argument with a spouse). Regularly leaves home without prompting. Rarely has difficulty initiating interactions with new people. If social interactions with community members, friends, and intimate partners are awkward or problematic, the individual has sufficient insight to make things right and it does not interfere with relationships. The individual has at least two reciprocal friends and several regular acquaintances whom they see in person at least 4x/month. Internet social contact may include gaming, text-based and verbal contact, face-to-face communication, and social media. |
|  | **Mild problems in social/interpersonal functioning** |
| **7** | **Original:** Some persistent mild difficulty in social functioning. Mild impairment present that is NOT just expectable reaction to psychosocial stressors (e.g., mild conflicts with peers, coworkers or classmates; difficulty resolving conflicts appropriately). Has some meaningful interpersonal relationships with peers (casual and/or close friends). Some difficulty developing or maintaining age-appropriate intimate relationships (e.g., multiple short-term relationships).  **Proposed:** Mild and transient social awkwardness or anxiety is present above and beyond what might be expected due to intermittent stressors (e.g. losing a relative, friend moving). Leaves the home without prompting 3-5x/week, not including attending school or fulfilling other role obligations. The individual has at least 2 reciprocal friendships and several regular acquaintances whom they see in person at least 1x/month. They are able to initiate these interactions. Can generally solve simple interpersonal problems with these friends and acquaintances. Internet social contact may include gaming and text-based and verbal with some face-to-face communication. |
|  | **Moderate impairment in social/interpersonal functioning** |
| **6** | **Original:** Moderate impairment in social functioning. Moderate impairment present (e.g., few close friends; significant but intermittent conflicts with peers, coworkers, or classmates). Moderate difficulty developing age-appropriate intimate relationships (e.g., infrequent dating). Occasionally seeks out others but will respond if invited by others to participate in an activity.  **Proposed:** Sometimes prefers to be alone (with the exception of family members). Leaves the home without prompting 3-5x/week, not including attending school or fulfilling other role obligations. Is known to be socially awkward or anxious and has difficulty initiating interactions with those he or she does not know well. The individual may have 1-2 reciprocal friendships, an intimate partner, and 1-3 acquaintances whom they see in person at least 1x/month. Internet social contact may include gaming, text-based and verbal contact, including livestream chatting, and social media. |
|  | **Serious impairment in social/interpersonal functioning** |
| **5** | **Original:** Serious impairment in social functioning. No close friends or intimate partner but has some casual social contacts (e.g., acquaintances, school/work friends only). Rarely seeks out others. Occasional combative or verbally argumentative behavior with peers. Beginning to withdraw from family members (e.g., does not initiate conversation with family, but will respond if addressed).  **Proposed:** Often alone (with the exception of contact with family members). Leaves the home without prompting 2-3x/week, when not attending school or fulfilling other role obligations. Socially awkward or extremely socially anxious. Has difficulty initiating interactions. The individual may have 1 reciprocal friendship or intimate partner, and several regular acquaintances whom they see in person at least 1x/month. Internet social contact may include gaming, text-based and verbal contact including livestream chatting, and social media. |
|  | **Major impairment in social and interpersonal functioning** |
| **4** | **Original:** Major impairment in social functioning. Serious impairment in relationships with friends or peers (e.g., very few or no friends, frequent conflicts with friends, or frequently avoids friends). Frequent combative or verbally argumentative behavior with peers. Infrequent contact with family members (e.g., sometimes does not respond to family or avoids family members).  **Proposed:** Socially isolated (with the exception of contact with family members). Leaves the home without prompting 2-3x/week, when not attending school or fulfilling other role obligations. Very socially inappropriate, awkward, or intensely socially anxious or depressed. Although they have no close friends or intimate partners, the individual has 1-3 acquaintances whom they see in person at least 1x/month, although they rarely initiate this contact in a formal way. Internet social contact may include gaming, text-based and verbal contact, and social media. |
|  | **Marginal ability to function socially** |
| **3** | **Original:** Marginal ability to function socially or maintain interpersonal relationships. Frequently alone and socially isolated. Serious impairment in relationships with all peers, including acquaintances. Few interactions with family members (e.g., often alone in room). Serious impairment in communication with others (e.g., avoids participating in most social activities).  **Proposed:** Typically, alone and socially isolated (with the exception of some contact with family members). Leaves the home without prompting infrequently (<2x/week), unless attending school or other role-related obligation. When interacting socially, may initiate this contact, but is either extremely socially inappropriate, awkward or intensely socially anxious or depressed. Has no true reciprocal friendships, regular acquaintances or intimate partners. Internet social contact is limited to gaming, infrequent text-based and verbal (e.g. phone calls) contact. May use social media. |
|  | **Inability to function socially** |
| **2** | **Original:** Unable to function socially or to maintain any interpersonal relationships. Typically, alone and socially isolated. Rarely leaves home. Rarely answers the phone or the door. Rarely participates in interactions with others at home or in other settings (eg, work, school).  **Proposed:** Almost always alone and socially isolated, with the exception of infrequent contact with family members. Rarely (<1x/week) leaves home to go out into the community unprompted other than to attend school (high school or secondary if still enrolled) School absences may be common. Rarely (< 1x/week) participates in interactions with others outside of home or school voluntarily. Internet social contact is limited to gaming, infrequent text-based communication 1-2x/month to mostly family members, with little or no use of social media. |
|  | **Extreme social isolation** |
| **1** | **Original:** Extreme social isolation. No social or family member contact at all. Does not leave home. Refuses to answer the phone or door.  **Proposed:** No social contact with individuals other than infrequent contacts with family members. No close friends. Doesn’t leave home—even when prompted. If in school (high school or secondary school), may be home schooled or take only online classes. If attending school on a traditional campus, avoids others when there. Internet social contact is limited to infrequent text-based (e.g. texting and e-mails) communication 1-2x/month to family members only, with no use of social media (e.g. FaceBook, Twitter, Instagram, SnapChat). |

**Results**

Table 1 of the Supplemental Materials provides a detailed description of the demographic characteristics of the participants and other clinical features.

| **Table 1.** Participants’ demographic characteristics. | | |
| --- | --- | --- |
|  | **Groups** | |
|  | **Non-autistic**  **(n = 66)** | **Autistic**  **(n = 66)** |
|  | Mean (SD) | Mean (SD) |
| Age | 18.7 (2.1) | 18.6 (2.1) |
| Age range | 16 – 24 | 16 – 24 |
| Sex (n, %) |  |  |
| Male | (53, 79%) | (55, 83%) |
| Female | (14, 21%) | (11, 17%) |
| Ethnicity (n, %) |  |  |
| White or Caucasian | 40, 62% | 46, 69% |
| Hispanic or Latino  African American | 4, 7%  6, 9% | 5, 8%  7, 11% |
| Asian | 13, 20% | 8, 12% |
| Pacific Islander or Native Hawaiian | 1, 2% | - |
| Mixed | 2, 3% | - |
| FSIQ | 110.1 (12.3) | 102.5 (13.6) |
| ADOS-2 | - | 14.7 (4.3) |
| ADOS-2 CSS | - | 7.91 (1.6) |
| SCQ | 2.9 (2.8) | 22.3 (2.8) |
| **FSIQ:** Full-Scale Intelligence Quotient; **ADOS-2**: Autism Diagnostic Observational Schedule-2; **CSS**: Calibrated Severity Score; **SCQ:** Social Communication Questionnaire. | | |

Between-group comparisons (autistic vs. non-autistic participants) of AF skills were conducted using a Mann-Whitney U test. Results revealed significant group differences (see table 2), with autistic participants demonstrating lower adaptive functioning skills with the average score being one standard deviation below that of non-autistic participants.

| **Table 2.** Between-group comparisons of adaptive functioning skills. | | | | | | |
| --- | --- | --- | --- | --- | --- | --- |
|  | **Group** | **n** | **Mean Rank** | **U** | **Z** | ***p-*value** |
| ABAS-3 PR |  |  |  |  |  |  |
| GAC | Non-Autistic  Autistic | 66  66 | 95.2  37.8 | 285.000 | -8.619 | <.001 |
| CONCEPTUAL | Non-Autistic  Autistic | 66  66 | 94.2  38.8 | 350.000 | -8.335 | <.001 |
| SOCIAL | Non-Autistic  Autistic | 66  66 | 95.3  37.7 | 280.500 | -8.656 | <.001 |
| PRACTICAL | Non-Autistic  Autistic | 66  66 | 93.4  39.7 | 406.000 | -8.072 | <.001 |
| ABAS-3 SR |  |  |  |  |  |  |
| GAC | Non-Autistic  Autistic | 66  66 | 91.0  41.9 | 556.500 | -7.397 | <.001 |
| CONCEPTUAL | Non-Autistic  Autistic | 66  66 | 89.1  43.9 | 686.000 | -6.821 | <.001 |
| SOCIAL | Non-Autistic  Autistic | 66  66 | 90.8  42.2 | 574.500 | -7.317 | <.001 |
| PRACTICAL | Non-Autistic  Autistic | 66  66 | 90.5  42.5 | 597.500 | -7.206 | <.001 |
| **ABAS:** Adaptive Behavior Assessment-3**; PR:** parent-report; **SR:** self-report; **GAC:** global adaptive composite.  The significance level at .05 | | | | | | |

**References**

Cornblatt, B. A., Auther, A. M., Niendam, T., Smith, C. W., Zinberg, J., Bearden, C. E., & Cannon, T. D. (2007). Preliminary findings for two new measures of social and role functioning in the prodromal phase of schizophrenia. *Schizophrenia bulletin*, *33*(3), 688–702. https://doi.org/10.1093/schbul/sbm029
